# Development of the ventricular myocardial trabeculae in *Scyliorhinus canicula* (Chondrichthyes): evolutionary implications

**DOI:** 10.1038/s41598-020-71318-x

**Published:** 2020-09-02

**Authors:** Miguel A. López-Unzu, Ana Carmen Durán, Cristina Rodríguez, María Teresa Soto-Navarrete, Valentín Sans-Coma, Borja Fernández

**Affiliations:** 1 Department of Animal Biology, Faculty of Science, University of Málaga, 29071 Málaga, Spain; 2 Instituto de Investigación Biomédica de Málaga-IBIMA, Málaga, Spain; 3 Instituto de Biotecnología y Desarrollo Azul-IBYDA, Málaga, Spain

**Keywords:** Embryology, Organogenesis, Evolution, Zoology, Ichthyology

## Abstract

The development of the ventricular myocardial trabeculae occurs in three steps: emergence, trabeculation and remodeling. The whole process has been described in vertebrates with two different myocardial structural types, spongy (zebrafish) and compact (chicken and mouse). In this context, two alternative mechanisms of myocardial trabeculae emergence have been identified: (1) in chicken and mouse, the endocardial cells invade the two-layered myocardium; (2) in zebrafish, cardiomyocytes from the monolayered myocardium invaginate towards the endocardium. Currently, the process has not been studied in detail in vertebrates having a mixed type of ventricular myocardium, with an inner trabecular and an outer compact layer, which is presumptively the most primitive morphology in gnathostomes. We studied the formation of the mixed ventricular myocardium in the lesser spotted dogfish (*Scyliorhinus canicula,* Elasmobranchii), using light, scanning and transmission electron microscopy. Our results show that early formation of the mixed ventricular myocardium, specifically the emergence and the trabeculation steps, is driven by an endocardial invasion of the myocardium. The mechanism of trabeculation of the mixed ventricular myocardium in chondrichthyans is the one that best reproduces how this developmental process has been established from the beginning of the gnathostome radiation. The process has been apparently preserved throughout the entire group of sarcopterygians, including birds and mammals. In contrast, teleosts, at least those possessing a mostly spongy ventricular myocardium, seem to have introduced notable changes in their myocardial trabeculae development.

## Introduction

The heart of *Scyliorhinus canicula*, as in other chondrichthyans, is anatomically composed of sinus venosus, atrium, ventricle and an outflow tract formed by a conus arteriosus and a bulbus arteriosus. All these segments, contained within the pericardial cavity, have myocardium in their walls, except for the bulbus arteriosus, which consists mainly of elastin and smooth muscle cells^1,2^. The ventricular myocardium of the jawed vertebrates (gnathostomes) can be classified into three main types: spongy or trabeculated, mixed and compact^3–9^. The spongy myocardium is characterized by the existence of prominent trabeculae and deep intertrabecular recesses, lined by the endocardium, which spread close to the epicardial surface of the ventricle. The mixed myocardium consists of a well-developed spongy layer, the spongiosa, covered by an outer layer of compact myocardium, the compacta, which is composed of densely arranged cardiac muscular fibers and is placed close to the epicardium. The compact type displays a prominent compact myocardial layer and a few trabeculae.

The process of development of ventricular trabeculae, or trabeculation, has been divided in three distinct steps: emergence, trabeculation and remodeling^10,11^. During emergence, small myocardial projections appear in the luminal side of the cardiac wall, which increase in length throughout the trabeculation step, to form a meshwork of trabeculae. Finally, the developing myocardium acquires its adult morphology during the remodeling step^10^.

Ventricular trabeculation has been studied in a limited number of gnathostomes. Current information on the issue refers to species such as the chicken (*Gallus gallus*) and the mouse (*Mus musculus*) that have a compact myocardium^10,12–15^ and the zebrafish (*Danio rerio*) that has a highly trabeculated myocardium (i.e. a thin external compact layer)^16–18^. Knowledge about the formation of the mixed type of myocardium is notably scarce, except for some brief accounts included in a general study of the heart development in the lesser spotted dogfish, *Scyliorhinus canicula*^19^. In the context of cardiovascular evolution, knowledge of the development of the mixed myocardium would be interesting, given that it is regarded as the most plesiomorphic myoarchitectonic condition in jawed vertebrates^20^.

Our study aims to fill this gap, characterizing the development of the myocardial trabeculae in the ventricle of *S. canicula*. The selection of the species adds in consideration previously reports revealing a mixed ventricular myocardium with a conspicuous compact layer in *S. canicula*^21^. In addition, its heart is believed to faithfully reflect the most plesiomorphic anatomical design of this organ in gnathostomes^20^. The species is oviparous, what facilitates obtaining embryos and determining their embryonic stage in the laboratory according to the criteria described by Ballard et al.^22^.

## Results

**Figure 1:**
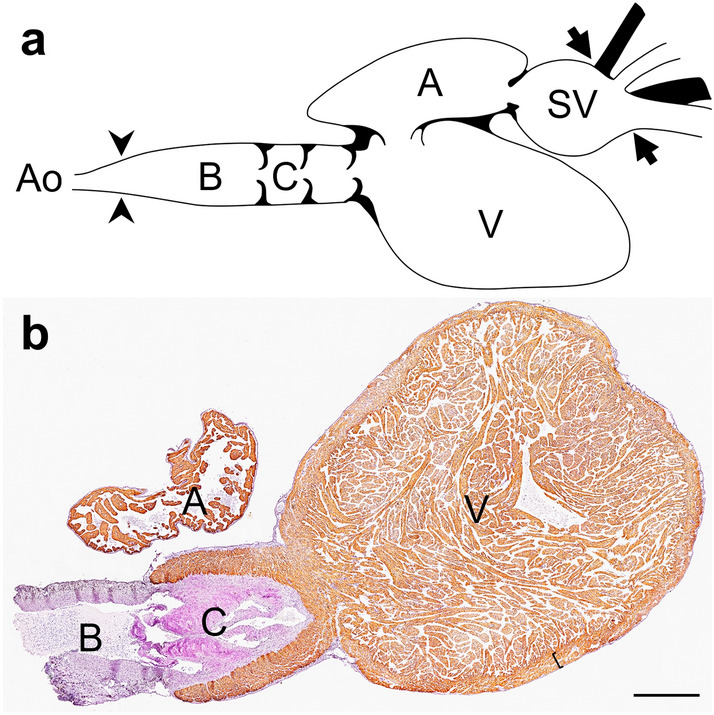
Heart of adult *Scyliorhinus canicula*. Schematic representation adapted from Ahnode [CC BY 3.0] (**a**). The arrowheads and arrows point to the anterior and posterior limits of the pericardial cavity, respectively. Sagittal section immunolabelled with MF20 antibody and counterstained with hematoxylin (**b**). The bracket delimits the compact layer of ventricular myocardium. A, atrium; Ao, ventral aorta; B, bulbus arteriosus; C, conus arteriosus; SV, sinus venosus; V, ventricle. Scale bar: 1 mm.

The present study includes embryos belonging to developmental stages 26–34, whose anatomical arrangement of the heart corresponded to that of the adult dogfish (Fig. 1a, b). All the observations were obtained from at least five specimens of each developmental stage studied, unless otherwise stated.

### Macroscopic findings

**Figure 2:**
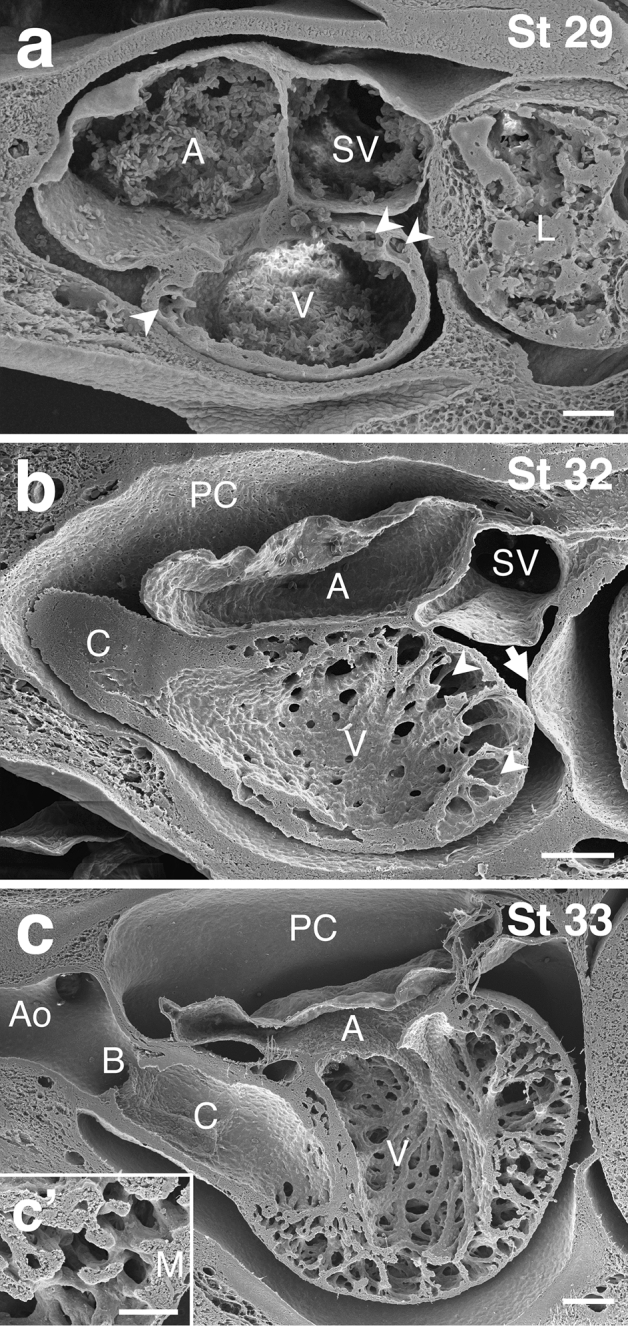
Scanning electron micrographs of the embryonic heart of *Scyliorhinus canicula*. Sagittal views of the heart at developmental stages 29 (**a**), 32 (**b**) and 33 (**c**, **c**′). The heart shows an ‘S’ shape. The sinus venosus (SV) and the atrium (A) occupy a dorsal position with regard to the ventricle (V) and the outflow tract, which consists of a conus arteriosus (C) and a bulbus arteriosus (B). In (**a**), the arrowheads point to intramyocardial cavities. In (**b**), the arrowheads mark the growing intramyocardial cavities. The arrow indicates the septum transversum. Note the presence of numerous perforations in the internal face of the ventricular wall. In (**c**), the ventricle shows conspicuous trabeculation. In (**c**′), perpendicular myocardial fascicles are anchored to the thin outer layer of myocardium (M). Ao, ventral aorta; L, liver; PC, pericardial cavity. Scale bars: 200 µm (**a**, **b**, **c**) and 30 µm (**c**′).

The trabeculation of the myocardium was studied using scanning electron microscopy in embryos at developmental stages 29, 32 and 33 (Fig. 2). At stage 29, the septum transversum was still incomplete and the sinus venosus remained connected to the liver (Fig. 2a). At stages 32 and 33, the septum transversum had already developed, forming a complete partition between the pericardial cavity and the peritoneal cavity (Fig. 2b, c).

At stage 29, the ventricular wall was thicker than that of the sinus venosus and atrium. The thickness was more apparent in the dorsal region of the ventricle, where some intramyocardial cavities were formed in the middle portion of the ventricular wall (Fig. 2a).

At stage 32 the intramyocardial cavities were enlarged and distributed throughout the ventricular wall. As a result, two separated myocardial layers, outer and inner, were distinguished. The outer myocardial layer was continuous around the entire ventricle and contacted the inner layer through small myocardial fascicles. The inner layer had perforations of various diameters connecting the intramyocardial cavities with the ventricular lumen (Fig. 2b). At stage 32, the wall of the sinus venosus and atrium did not show an apparent increase in thickness.

At stage 33, the ventricular myocardium showed a marked degree of trabeculation, acquiring a sponge appearance (Fig. 2c). The trabeculae, which were anchored to the thin outer layer of myocardium, formed a meshwork that occupied most of the ventricular lumen (Fig. 2c′). The walls of the sinus venosus and atrium showed no significant change compared to stage 32.

### Histomorphological findings

**Figure 3:**
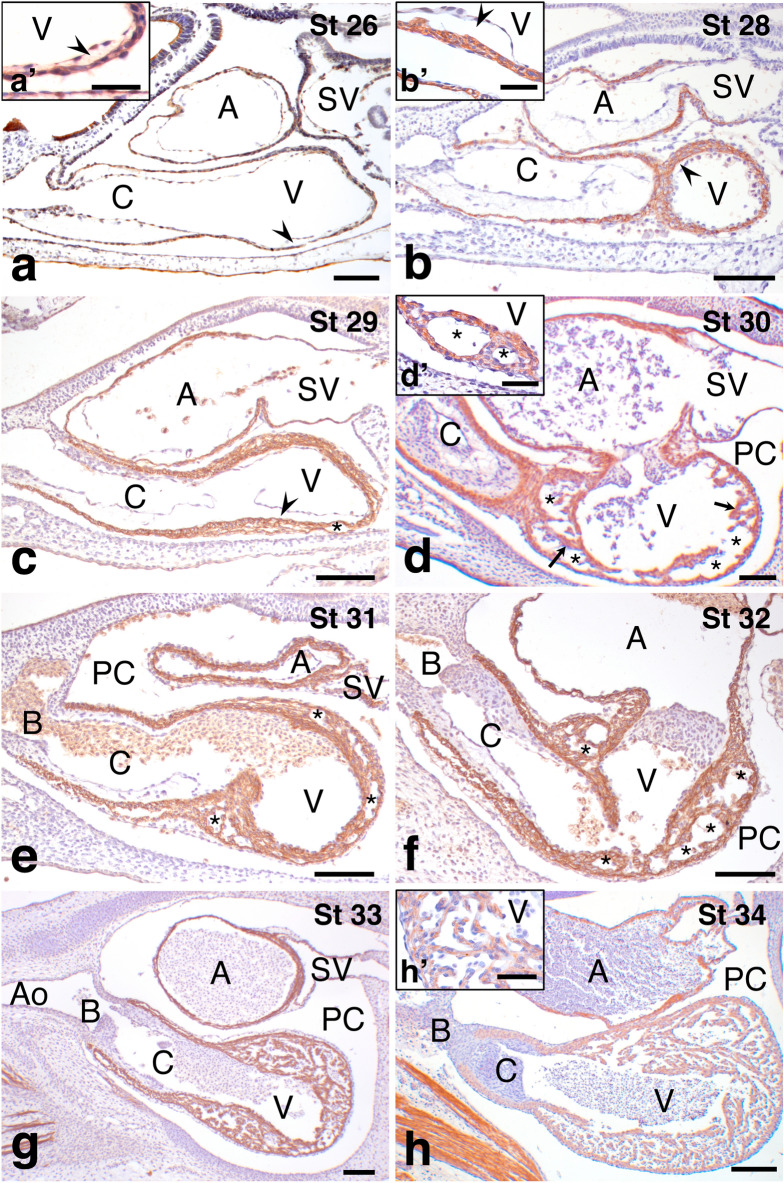
Sagittal sections of the developing heart of *Scyliorhinus canicula* immunolabeled with A4.1025 antibody and counterstained with hematoxylin. (**a**) Stage 26. The heart consists of four A4.1025-positive segments: sinus venosus (SV), atrium (A), ventricle (V) and conus arteriosus (C). The inset (**a**′) shows the endocardium and the monolayered myocardium separated by the cardiac jelly (arrowhead). (**b**) Stage 28. The ventricular myocardium, which is thicker than the rest of the myocardium, has up to three densely arranged myocyte layers (**b**′). (**c**) Stage 29. Small spaces (asterisk) appear in the middle zone of the ventricular myocardium. (**d**) Stage 30. The cardiac jelly has disappeared. The intramyocardial spaces become larger, forming cavities (asterisks) in the ventricular wall, which are delimited by radial myocardial fascicles or trabecular ridges (arrows). The cavities are coated by endocardium, as shown in the inset (**d**′). (**e**) Stage 31. Two myocardial layers, inner and outer, separated by conspicuous cavities (asterisk), are distinguishable in the ventricle. (**f**). Stage 32. The cavities are wider, and the ventricular wall is thicker. (**g**) Stage 33. The myocardial trabeculae of the ventricle become more complex, forming a meshwork. (**h**) Stage 34. A4.1025 labelling decreases in both the ventricle and conus. The trabecular meshwork resembles that of the adult. The developing trabeculae are lined by endocardium and anchored to the prospective compact layer (**h**′). B, bulbus arteriosus; PC, pericardial cavity. Scale bars: 100 µm (**a**–**h**) and 50 µm (**a**′, **b**′, **d**′, **h**′).

The earliest embryos studied by immunohistochemical techniques belonged to the stage 26. The heart was S-shaped in lateral view (Fig. 3a). Its walls were composed of the endocardium and a monolayered myocardium, separated each other by the cardiac jelly, and externally lined by the developing epicardium (Fig. 3a′). The myocardium showed positive immunoreactivity against the anti-Myosin heavy chain (MyHC) antibody A4.1025 (Developmental Studies Hybridoma Bank). At stage 28, the myocardium, which was thinner in the ventral part of the ventricle (Fig. 3b), presented up to three densely arranged cardiomyocyte layers (Fig. 3b′).

In the following stage, stage 29 (Fig. 3c), the number of myocardial layers did not increase in the ventricle and the cardiac jelly remained thick. However, several cavities were formed in the middle portion of the ventricular wall, which did not exist in the myocardium of the other cardiac segments.

At stage 30 (Fig. 3d), the intramyocardial spaces in the ventricle markedly increased in number and size, whereas the cardiac jelly became thinner. Two parallel myocardial layers, outer and inner, were well developed. They were interconnected by small perpendicular myocardial fascicles (Fig. 3d′). The outer myocardial layer was continuous, whereas the inner layer was interrupted in numerous places, connecting the interventricular cavities with the ventricular lumen.

During stages 31 and 32, the myoarchitecture of the ventricular wall did not significantly differ from the previous stage (compare Fig. 3e, f). However, the wall of both atrium and ventricle had increased in thickness.

At the next stage, the wall of the ventricle was even thicker, and the outer myocardial layer was internally coated by a meshwork of trabeculae of myocardial cells (Fig. 3g). The meshwork was in continuity with the inner layer of myocardium, which remained perforated, establishing thereby a connection between the myocardial recesses and the lumen of the ventricle. Then, at stage 34 (Fig. 3h), the ventricular myocardium showed a conspicuous trabeculation (Fig. 3h′) and a decrease in the intensity of the MyHC staining. In contrast, the atrial myocardium was labelled strongly.

### Ultrastructural findings

**Figure 4:**
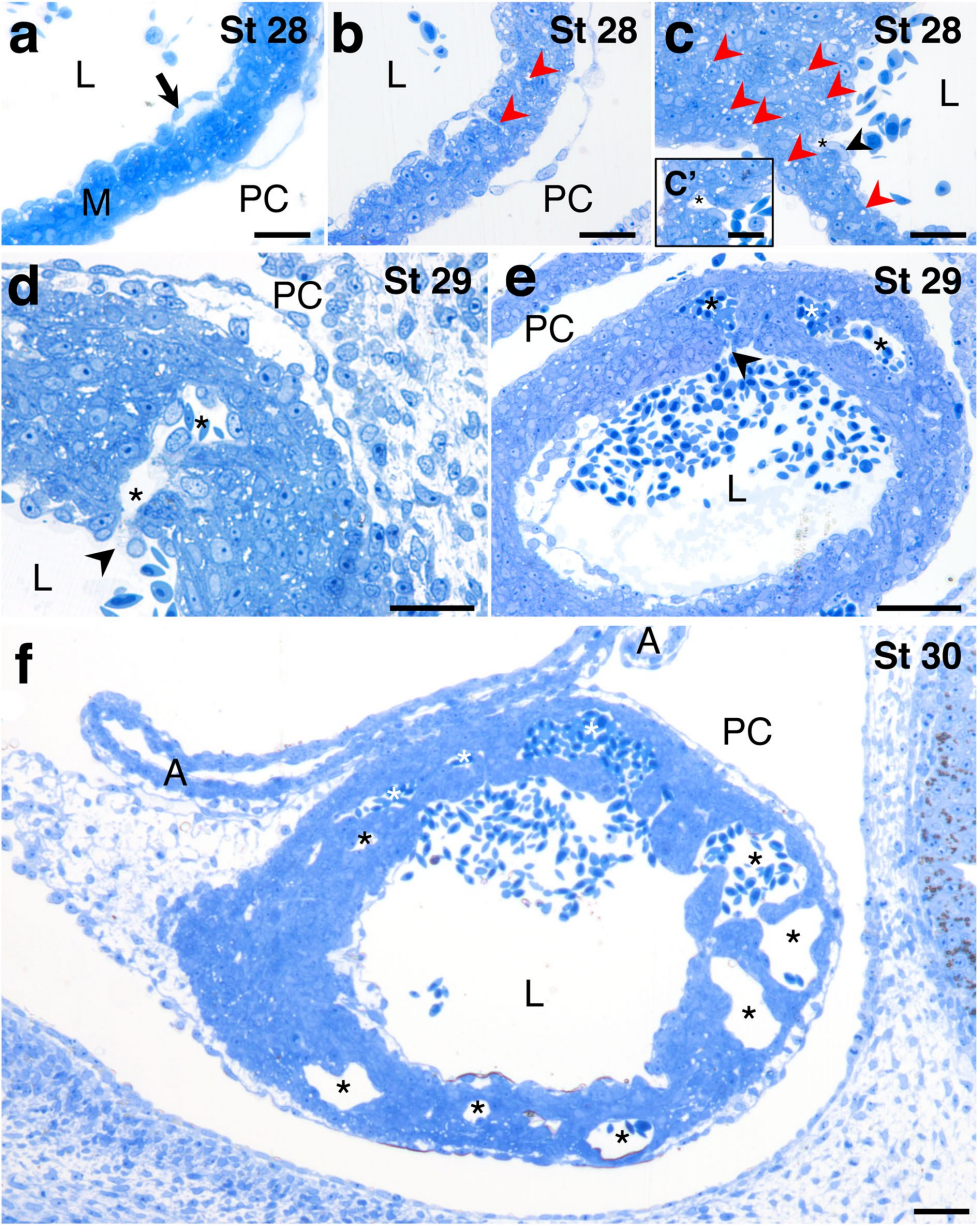
Sagittal semithin sections of the heart of *Scyliorhinus canicula* embryos at developmental stages 28 (**a**–**c**), 29 (**d**, **e**) and 30 (**f**) stained with toluidine blue. At the beginning of stage 28 (**a**) the ventricular myocardium (M) has up to three layers of cardiomyocytes. Strands of endocardial cells can be locally seen where the cardiac jelly in regression still persists (arrow). Halfway through the stage 28 (**b**), small intercellular spaces (red arrowheads) appear in the ventricular myocardium. At the end of stage 28 (**c**) the endocardium focally invades (black arrowhead) and coats the myocardium. Intercellular spaces (red arrowheads) increase in number. At the beginning of stage 29 (**d**) intramyocardial cavities (asterisks) develop and connect (black arrowhead) with the ventricular lumen. At the end of the stage 29 (**e**), blood cells fill the intramyocardial cavities (asterisks). At stage 30 (**f**), as myocardial trabeculae develop, cavities (asterisks) become well defined intertrabecular spaces connected with the ventricular lumen. A, atrium; L, ventricular lumen; PC, pericardial cavity; V, ventricle. Scale bars: 50 µm (**a**–**f**) and 25 µm (**c**′).

**Figure 5:**
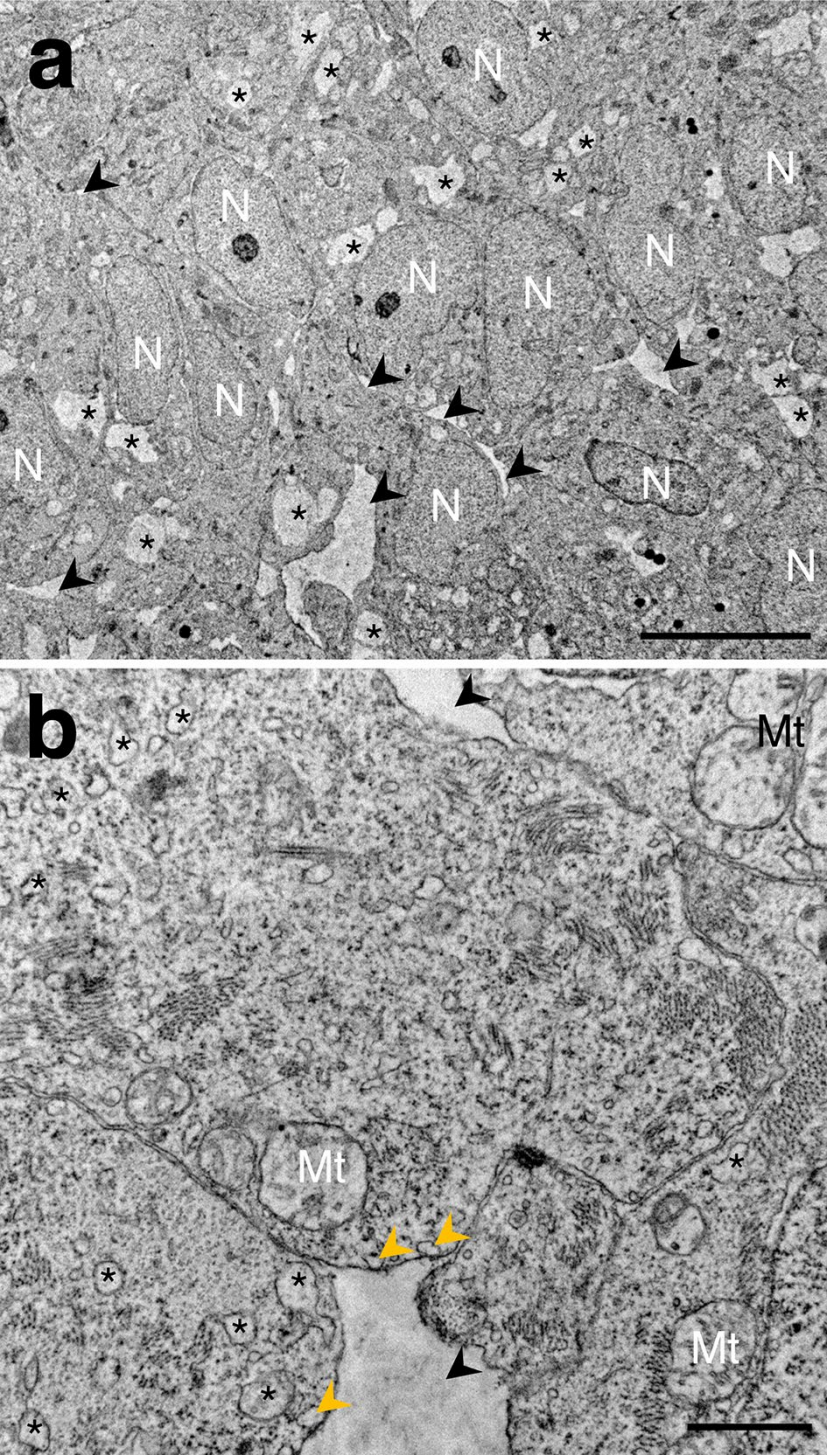
Transmission electron micrographs of the ventricular myocardium of *Scyliorhinus canicula* at developmental stage 28. (**a**) The cardiomyocytes contain small vesicles (asterisks) and variable sized extracellular spaces (arrowheads). (**b**) A closer view to these spaces confirms their intercellular position. Cardiomyocytes surrounding the intramyocardial spaces contain vesicles (asterisks), some of which (yellow arrowheads) are in contact with the cell membrane limiting the extracellular spaces (black arrowheads). Mt, mitochondria; N, nucleus. Scale bars: 10 µm (**a**) and 1 µm (**b**).

Semithin sections of ventricles from developmental stages 28–30 were used for a detailed assessment of the trabecular emergence (Fig. 4). At the beginning of the process (stage 28), the myocardium showed a compact appearance, while the cardiac jelly, between the myocardium and the endocardium, was in regression. Only some short, scattered strands of endocardial cells were separated from the myocardium by cardiac jelly (Fig. 4a). During this developmental phase, the myocardium was less compact. Small spaces had appeared in the myocardium (Fig. 4b), becoming larger over time. At the end of stage 28, the endocardium began to invade and coat the myocardium, specifically at places where the myocardial intercellular spaces had developed. The progressive invagination of the endocardium into the myocardium resulted in the coverage of the intramyocardial spaces formed in the previous stage by endocardial cells (Fig. 4c). Transmission electron microscopy allowed a more accurate assessment of the spaces, which consisted of intercellular spaces and small intracellular vesicles, both not electrodense (Fig. 5). Cardiomyocytes surrounding the intercellular spaces contained numerous small vesicles (Fig. 5a), some of them located next to the cell membrane facing the space (Fig. 5b).

At stage 29, the intramyocardial cavities were fully covered with endocardial cells (Fig. 4d). The cavities were in continuity with the ventricular lumen by narrow channels through which blood flow was established. (Fig. 4d, e). At stage 30, as myocardial trabeculae develop, cavities become well defined intertrabecular spaces (Fig. 4f).

## Discussion

Our report seems to be the first to provide an extensive description of the development of myocardial trabeculae in a ventricle with mixed myocardium, i.e., with a thick compacta, specifically in *S. canicula*, a representative of chondrichthyans. Taken together, our results show that the formation of trabeculae in this type of myocardium takes place in three steps: emergence, trabeculation and remodeling. In *S. canicula*, these steps are more similar to those described in animal models with compact myocardium than in those with spongy myocardium.

**Figure 6:**
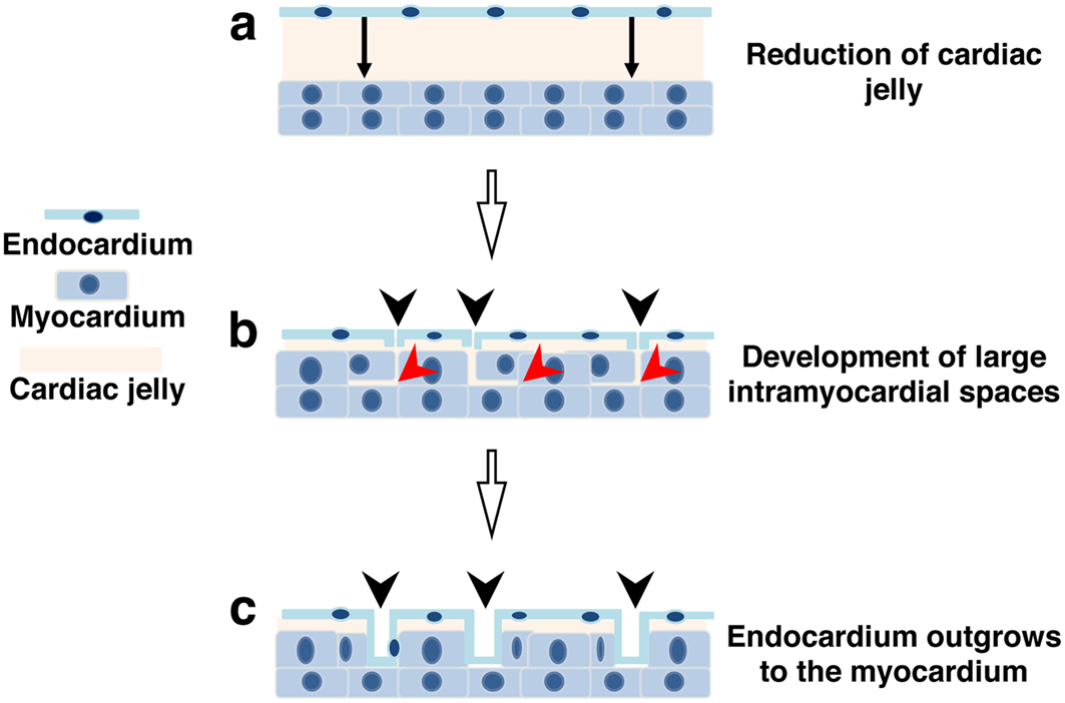
Schematic representation of the emergence step of the mixed ventricular myocardium development. (**a**) Decrease in thickness of the cardiac jelly. (**b**) Development of intramyocardial spaces (red arrowheads) and focal contact of the endocardial cells with the myocardium (black arrowheads). (**c**) Endocardial cell invasion and coating of the myocardium.

The emergence occurs during stage 28. At the beginning of this stage, the heart is composed of three cellular layers, the external epicardium, the internal endocardium and the intermediate myocardium, the two latter separated by the cardiac jelly. The ventricular myocardium consists of two to three sheets of densely arranged MyHC positive cells. Then, small intramyocardial spaces between cardiomyocytes appear. Concomitantly, the cardiac jelly between the myocardium and the endocardium becomes thinner, causing occasional contacts between the two layers. Thereafter, endocardial cells invade the intramyocardial spaces, forming several small recesses that connect the intramyocardial spaces with the ventricular lumen (Fig. 6).

The trabeculation step occurs during a longer developmental period than the emergence. The myocardium increases in thickness, and the small intramyocardial spaces grow in size becoming conspicuous cavities. This observation is in agreement with that of Muñoz-Chápuli et al.^19^, who briefly referred to the origin of the ventricular trabeculae in a general study of the heart development in *S. canicula*. Then, the innermost sheet of myocardial cells becomes discontinuous, which allows wide connections between the ventricular lumen and the intramyocardial cavities. The connections increase gradually in number and size and get filled with blood from the ventricular lumen. Concomitantly, several intramyocardial cavities coalesce in the middle of the myocardium connected by myocardial fascicles. Similar fascicles have been previously described in other vertebrate groups and named as trabecular ridges or sheets. In *S. canicula* however, the ridges are oriented radially, different to amphibians, reptiles, birds and mammals^10,23^*,* in which they show a dorsoventral alignment, what was suggested to be related with the formation of the interventricular septum^10,23,24^. The remodeling step starts at the end of the developmental stage 33. The inner myocardial layer becomes a meshwork of cylindrical trabeculae, which are anchored to the outer myocardial layer. The trabeculae increase in size, adopting progressively the irregular shapes that can be seen in the spongy or trabeculated layer of the adult mixed myocardium. At stage 33, the outer myocardial layer remains thin, indicating that it mainly develops after hatching, which in *S. canicula* occurs at the end of stage 34.

At stage 34 the intensity of the MyHC (A4.1025) antibody labeling decreases in the ventricular myocardium. In this regard, it should be noted that the A4.1025 antibody has been successfully used to detect differences in the location of MyHC isoforms in the myocardium of adult specimens of *S. canicula*^21^. In this animal model, the fast-twitch isoforms, specifically MYH2 and MYH6 show a higher affinity for the antibody than the slow-twitch isoforms (MYH7 and MYH7B). Thus, the present immunohistochemical data corresponding to developmental stage 34 suggest that before hatching there is a MyHC isoform shift from fast-twitch to slow-twitch isoforms in the cardiac ventricle of the dogfish.

The early trabeculation steps of the myocardium in *S. canicula* are similar to those described in chicken^12^ and mouse^15^. In the three animal models, before the trabeculation, the ventricular myocardium is composed of more than a single layer of cardiomyocytes. The emergence in these species starts with the formation of intercellular spaces, which grow and coalesce to finally disengage the myocardium in an inner and an outer layer^12,15^. The present study shows that the same process occurs during the cardiac development of the dogfish. Furthermore, our transmission electron microscopy results show numerous myocardial intracellular vesicles close to the cellular membranes that delimit the intercellular spaces. According with this, we propose that the cardiomyocytes of the developing ventricle secrete the extracellular material which fills these intramyocardial spaces.

As pointed out by Kruithof et al.^15^, the separation of the myocardium in two layers is achieved when the endocardial cells invade the inner layer. This idea is also consistent with our results, which show that when the cardiac jelly decreases in thickness, the endocardial cells focally contact and protrude into the myocardium through the intramyocardial spaces, which are progressively coated by the invading cells to form bigger cavities. In addition, our observations also coincide with recent findings obtained in mouse embryos during trabeculation stages, in which endocardial cells first contact the myocardium through cardiac jelly via endocardial columns or ‘touchdowns’^25^.

The process of trabeculation in chondrichthyans and tetrapods diverges from that in the zebrafish^16–18^, in which the emergence occurs as an ‘active invagination’ of the cardiomyocytes towards the ventricular lumen^17^. This is consistent with the observations of Liu et al.^16^ and Staudt et al.^18^, who showed that in this animal model, the trabeculation of the ventricular myocardium is basically driven by delamination of the cardiomyocytes of the primary myocardial layer.

Classically, it was assumed that the ventricle of the plesiomorphic vertebrate heart was composed exclusively of spongy myocardium, and that the mixed and compact myocardial types appeared independently in different groups during evolution^26–28^. However, later work provided evidence that the mixed myocardium is the plesiomorphic state of the trait in gnathostomes, whereas both the spongy and compact myocardia are derived conditions^9,20^. On this basis, it has been proposed that the absence of a myocardial compact layer in the ventricle of several actinopterygian and sarcopterygian species should be regarded as a result of its repeated loss during evolution^29^. Loss of compact myocardium might be linked to the lack of coronary circulation in some cardiac segments^30^. Likewise, the presence of mostly compact myocardium in other species may be due to the allometric growth of the external myocardial layer, namely, the compacta.

The present results suggest that trabeculation of mixed myocardium takes place by means of endocardial cell invasion of the myocardium during the emergence step of *S. canicula*. This mechanism is similar to that described in tetrapods, specifically in birds and mammals, but not in teleosts, in which the process of emergence takes place through a different mechanism, i.e., myocardial invasion of the cardiac jelly and protrusion towards the lumen. Notably, zebrafish is the only teleost model in which trabeculation has been studied. Thus, our results suggest that the mechanism that governs the formation of the myocardial trabeculae has been preserved from chondrichthyans to sarcopterygians, including birds and mammals, and must be regarded as the plesiomorphic condition. Accordingly, the mechanism of myocardial trabeculae development in zebrafish must be regarded as a derived trait.

Under this assumption, birds and mammals and other sarcopterygians, as coelacanths^31^ would have retained and expanded a conspicuous vascularized compacta, while in dipnoans^32^, amphibians and several reptiles^33^, the compact myocardial layer would have been markedly reduced in thickness. Birds^10^ and mammals^34,35^ could have retained a very thin layer of spongy myocardium as a sort of reminiscence, while the compacta became predominant in the ventricle. In these groups, the development of compact myocardium and blood supply through coronary arteries could be associated events, given that the growth of the compact ventricular myocardium starts when the coronary system development initiates^10^. In this regard, it will be interesting to elucidate the mechanism of emergence in sarcopterygians with ventricles mainly composed of spongy myocardium, like amphibians or dipnoans.

In early groups of extant actinopterygians, the myoarchitecture of the ventricle is variable. Polypteriforms^36,37^ and ammiiforms^36^ have only spongy myocardium in the ventricle, whereas lepisosteiforms^36^ and acipenseriforms^38^ possess mixed myocardium with a thick compact myocardium. It remains to be elucidated whether the spongy and mixed myocardia of early actinopterygians develop as a consequence of the plesiomorphic or the derived developmental mechanism.

Some teleost species, even belonging to phylogenetically distant groups (orders) have a ventricle with a high proportion of compact myocardium^36,39–42^. In other cases, as zebrafish, the myocardium is mostly trabeculated^36^. The current concept is that the compact layer has almost disappeared in most teleost species^9,20^. The present results fit with this evolutionary scenario, so that, at some point during the evolution of actinopterygians, a new mechanism of trabeculation should have appeared. This novelty might be related to the whole-genome duplication that lead to the explosive radiation of teleosts^43–45^. Most of the duplicated genes underwent either loss, or acquired redundant function or sub-functionalization^46,47^. The process probably led to the appearance of a variety of new molecular mechanism affecting the embryogenesis of teleosts^48,49^. In this context, it should be noted that the zebrafish, the most studied fish model, belongs to the cypriniforms, which occupy a phylogenetical position away from the representatives of most ancient actinopterygians^50^. The question is how and to what extent the genomic reorganization altered the development program of the ventricular myocardium in teleosts, producing its divergence from that of the group of chondrichthyans. Therefore, further embryological studies with additional actinopterygians species, preferentially displaying different degrees for ventricular myocardium compaction, are needed to assess if the process of trabeculation observed in zebrafish, often seen as the representative of the whole clade, and how this process evolved across teleosts.

In conclusion, our present results shows that the early formation, specifically the emergence and the trabeculation, of the mixed ventricular myocardium in the lesser spotted dogfish (*Scyliorhinus canicula*) is similar to that in birds and mammals. The mechanism is driven by an endocardial invasion of the myocardium. This process diverges from that described in the zebrafish, in which the emergence occurs as an ‘active invagination’ of the cardiomyocytes towards the ventricular lumen.

The formation of the ventricular myocardium in chondrichthyans is the one that best reproduces how this developmental process was established from the beginning of the gnathostome radiation. Thus, the present results suggest that, in essence, the process has been preserved throughout the entire group of sarcopterygians, including birds and mammals. In contrast, teleosts, at least those possessing a mostly spongy ventricular myocardium, seem to have introduced notable changes in their myocardial development program.

## Methods

### Animals

The material examined consisted of 101 embryos and two adult specimens of *Scyliorhinus canicula*. Fertilized eggs were obtained from adult females collected by commercial fishing vessels in Caleta de Vélez, Málaga, Spain (Western Mediterranean). All the procedures were approved by the Spanish funding agency and performed at the University of Málaga, which is a licensed establishment under the European and Spanish guidelines on the protection of animals used for scientific purposes. In addition, all methods were performed in accordance with the European and Spanish guidelines and regulations (Directive 86/609/EEC on the protection of animals used for scientific purposes). The adult animals were already dead when they were collected from the boats. Eggs were transferred to the laboratory and maintained in perforated cages placed in a tank with seawater. The development of the embryos of *S. canicula* could be well followed by viewing them through the transparent walls of the egg capsule. To obtain embryos, eggs were opened and the embryos were carefully removed and over-anaesthetized with 0.04% MS-222 (Sigma-Aldrich Chemical Co., Poole, UK) in elasmobranch buffer (16.38 g L^−1^ NaCl, 0.89 g L^−1^ KCl, 1.11 g L^−1^ CaCl_2_, 0.38 g L^−1^ NaHCO_3_, 0.06 g L^−1^ NaHPO_4_, 21.6 g L^−1^ urea, pH 7.2). The degree of development of the embryo was estimated under the stereomicroscope according to the morphological stages established by Ballard et al.^22^. The embryos included in the present study belonged to stages 26 (n = 5), 28 (n = 9), 29 (n = 12), 30 (n = 12), 31 (n = 10), 32 (n = 16), 33 (n = 24) and 34 (n = 13).

### Scanning electron microscopy

The methods used for scanning electron microscopy have been described elsewhere^51,52^. Six embryos at developmental stages 29, 32 and 33 were fixed in 4% paraformaldehyde in elasmobranch buffer or methanol/acetone/water (2:2:1). Then, the embryos were dehydrated in a graded series of ethanol and embedded in paraffin (Histosec, Merck KGaA; Darmstadt, Germany). The embryos were sagittally oriented and cut until obtaining a suitable image of the whole heart at the level of its medial plane. Then, the paraffin block was dewaxed in xylene. Finally, the heart was dehydrated in increasing concentrations of ethanol, dried at the critical point and gold sputter coated. Observations were made using a Jeol JSM-840 scanning electron microscope (Jeol, Tokyo, Japan), operated at 5, 10 or 15 kV.

### Immunohistochemical techniques

The immunohistochemical protocol was the same as the one described in López-Unzu et al.^21^. Eighty-three dogfish embryos at developmental stages 26–34 were fixed in 4% paraformaldehyde in elasmobranch buffer or methanol/acetone/water (2:2:1). Then, the embryos were dehydrated in a graded series of ethanol and embedded in paraffin (Histosec, Merck KGaA; Darmstadt, Germany). Serial sections of the hearts cut sagittally at 8 μm were dewaxed, hydrated and washed in Tris-PBS (TPBS, pH 7.8). Some sections were counterstained with hematoxylin staining. The sections were observed with a Leica DMSL light microscope or with an Olympus VS120 virtual microscopy slide scanning system (Olympus, Tokyo, Japan) equipped with the VS-ASW software (Olympus, Tokyo, Japan) and viewed using the free of charge software OlyVIA (Informer Technologies, Inc., Walnut, CA, USA).

The first antibody was the monoclonal anti-MyHC A4.1025 (Cat# A4.1025, RRID: AB_528356; Developmental Studies Hybridoma Bank, University of Iowa) and MF20 (Cat# MF-20, RRID: AB_2147781; Developmental Studies Hybridoma Bank, University of Iowa). They were used at a dilution of 1:200 and 1:20 of both supernatants, respectively.

### Semithin sections

The heart together with the pericardium of eight embryos at developmental stages 28, 29 and 30 were fixed in 3% glutaraldehyde in elasmobranch buffer (pH 7.4). They were rinsed in elasmobranch buffer, post-fixed in 1% osmium tetroxide (Sigma-Aldrich Chemical Co., Poole, UK), dehydrated in graded acetone concentrations, rinsed in propylene oxide (Sigma-Aldrich Chemical Co., Poole, UK), and embedded in Araldite epoxy resin (Electron Microscopy Sciences, Hatfield, PA, US). Serial sections sagittally cut at 1 μm were obtained by a Leica EM UC7 ultramicrotome (Leica, Wetzlar, Germany). The sections were stained with 0.2% toluidine blue and observed with a Leica DMSL light microscope.

### Transmission electron microscopy

The heart together with the pericardium of four embryos at developmental stages 28, 29 and 30 were fixed and post fixed as was done for the semithin sections. Then they were dehydrated in graded acetone concentrations, rinsed in uranyl acetate, and embedded in Araldite epoxy resin (Electron Microscopy Sciences, Hatfield, PA, US).

The sections were obtained using a Leica EM UC7 ultramicrotome (Leica, Wetzlar, Germany). The 60 nm thick sections were observed with a Jeol JEM-1400 transmission electron microscope (Jeol, Tokio, Japan) equipped with a Gatan ES1000W camera (Gatan-Roper Technologies, Lakewood Ranch, FL, US) operated at 80 kV.

## Data Availability

The datasets used and analysed during the current study are available from the corresponding author on reasonable request.
